# Phylogenetic discordance of human and canine carcinoembryonic antigen (CEA, CEACAM) families, but striking identity of the CEA receptors will impact comparative oncology studies.

**DOI:** 10.1371/currents.RRN1223

**Published:** 2011-03-31

**Authors:** Marlene Weichselbaumer, Michael Willmann, Martin Reifinger, Josef Singer, Erika Bajna, Yuriy Sobanov, Diana Mechtcherikova, Edgar Selzer, Johann G. Thalhammer, Robert Kammerer, Erika Jensen-Jarolim

**Affiliations:** ^*^Clinic for Internal Medicine & Infectious Diseases, Dept. 4, University of Veterinary Medicine Vienna and Department of Pathophysiology and Allergy Research, Center of Pathophysiology, Infectiology & Immunology, Medical University of Vienna, Austria; ^†^Clinic for Internal Medicine & Infectious Diseases, Dept. 4, University of Veterinary Medicine Vienna, Austria; ^‡^Institute of Pathology and Forensic Veterinary Medicine, Department of Pathobiology, University of Veterinary Medicine, Vienna, Austria; ^§^Department of Pathophysiology and Allergy Research, Center of Pathophysiology, Infectiology & Immunology, Medical University of Vienna, Austria; ^††^Department Radiation Oncology, Medical University Vienna, Austria and ^§§^Friedrich-Loeffler-Institute (FLI) Institute of Immunology Paul-Ehrlich-Strasse 28 72076 Tübingen Germany

## Abstract

Comparative oncology aims at speeding up developments for both, human and companion animal cancer patients. Following this line, carcinoembryonic antigen (CEA, CEACAM5) could be a therapeutic target not only for human but also for canine (Canis lupus familiaris; dog) patients. CEACAM5 interacts with CEA-receptor (CEAR) in the cytoplasm of human cancer cells. Our aim was, therefore, to phylogenetically verify the antigenic relationship of CEACAM molecules and CEAR in human and canine cancer.

Anti-human CEACAM5 antibody Col-1, previously being applied for cancer diagnosis in dogs, immunohistochemically reacted to 23 out of 30 canine mammary cancer samples. In immunoblot analyses Col-1 specifically detected human CEACAM5 at 180 kDa in human colon cancer cells HT29, and the canine antigen at 60, 120, or 180 kDa in CF33 and CF41 mammary carcinoma cells as well as in spontaneous mammary tumors. While according to phylogenicity canine CEACAM1 molecules should be most closely related to human CEACAM5, Col-1 did not react with canine CEACAM1, -23, -24, -25, -28 or -30 transfected to canine TLM-1 cells. By flow cytometry the Col-1 target molecule was localized intracellularly in canine CF33 and CF41 cells, in contrast to membranous and cytoplasmic expression of human CEACAM5 in HT29. Col-1 incubation had neither effect on canine nor human cancer cell proliferation. Yet, Col-1 treatment decreased AKT-phosphorylation in canine CF33 cells possibly suggestive of anti-apoptotic function, whereas Col-1 increased AKT-phosphorylation in human HT29 cells. We report further a 99% amino acid similarity of human and canine CEA receptor (CEAR) within the phylogenetic tree. CEAR could be detected in four canine cancer cell lines by immunoblot and intracellularly in 10 out of 10 mammary cancer specimens from dog by immunohistochemistry. Whether the specific canine Col-1 target molecule may as functional analogue to human CEACAM5 act as ligand to canine CEAR, remains to be defined. This study demonstrates the limitations of comparative oncology due to the complex functional evolution of the different CEACAM molecules in humans versus dogs. In contrast, CEAR may be a comprehensive interspecies target for novel cancer therapeutics.

 

## Introduction 

Comparative oncology trials represent a novel preclinical infrastructure for speeding up the development of anticancer therapeutics [1] [2]. In contrast to known preclinical animal models, comparative studies take advantage of spontaneously occurring cancers, for instance in dogs (Canis lupus familiaris) [3]. Following specific guidelines [4], these studies also assess the response of the veterinary patients to novel drugs in terms of clinical benefit [5] [6] [7]. Mammary neoplasms are the most common tumors in female dogs [8], with incidence depending on the population studied and varying from 205 per 100,000 [9] to 111 per 10,000 female dogs between 3 and 10 years of age [10]. 30 to 50% of all canine mammary tumors are malignant and 50 to 75% of these recur or metastasize within 1 to 2 years [11] [12]. For these reasons more effective therapy strategies are urgently needed for companion animals, at the same time their disease mirrors the settings of human oncology patients. 

Especially immunological targeting of cancer for diagnosis and therapy is much more advanced in humans [13] [14]. Therefore, we suggest that careful, comparative evaluation of most important tumor targets should be undertaken to increase the basic knowledge for comparative studies. 

An important example for a potential immunological cancer target is carcinoembryonic antigen (CEACAM5), which represents a classical tumor marker and is routinely exploited for diagnosis in man. In its soluble form CEACAM5 binds to the carcinoembryonic antigen receptor (CEAR), a member of the heterogeneous nuclear ribonucleoproteins (hnRNP) family, offering the likely potential to deliver prometastatic signals to neoplastic cells [15] [16]. In many types of human cancers CEACAM5 is specifically expressed as a cell surface glycoprotein exhibiting several functions like regulating intercellular adhesion, differentiation and anoikis, cell polarization and tissue architecture [17]. In humans the level of overexpression determines tumor cell migration, invasion and formation of distant metastases and may therefore be a measure of malignancy [18]. High levels of CEACAM5 expression are found in human patients in 80 - 90% of all colorectal cancers, in up to 50% of all breast and lung cancers and in about 15% of gastrointestinal and pancreatic carcinomas as well as in many other epithelial cancers [19]. It has, therefore, been recognized as a clinically relevant target for passive and active immunotherapy. CEA has been the focus of multiple preclinical and clinical human studies using antibodies or fragments for targeted therapy [20] [21] [22] [23] [24] [25] or vaccines for active stimulation of B- and T-cell immunity [26] [27] [28] [29] [30], including one of our own studies [31]. 

Preclinical vaccination studies based on human CEACAM5 have been pursued in mice and also in dogs [29] [32] [33]. These efforts were based on the fact that carcinoembryonic antigen had been previously proposed as tumor marker in several canine immunohistochemical studies using anti-human CEACAM5 antibodies [34] [35] [36] such as Col-1 [37]. Monoclonal antibody Col-1 is originally known for its excellent diagnostic properties for the detection of premalignant and malignant lesions and metastases in humans [38] [39] [40] [41]. This antibody also served as the model for anti-idiotypic antibody production to elicit specific immune responses [42] and it has recently been humanized for safer clinical application as radio-immunoconjugate in human patients [43]. 

Thus CEACAM5 or Col-1 based diagnostic and therapeutic strategies in canine patients are further developed without precise knowledge of the target molecule. Human CEACAM5 belongs to the CEACAM1-related molecules within the CEA gene family. Although no unambiguous orthologous gene to human CEACAM5 was found in the dog genome [44] our phylogenetic analysis provides evidence that molecules related to the canine CEACAM1-family show the highest similarity to human CEACAM5 (Fig. 1). However, the lack of the sequence of canine CEACAM5 in the database is not *a priori* proof that the gene is absent. 

Therefore, it is to date still unclear whether CEACAMs are relevant targets for comparative studies. Based on the current knowledge of the phylogenetic associations, we aimed here to verify the antigenic relationship of human and canine CEACAMs as well as of CEAR. 

## Materials and Methods

***Patients material and Immunohistochemistry***

The study was conducted with tissue samples from 30 canine mammary tumor patients. Tissue specimens of canine mammary glands were obtained either during diagnostic biopsy or during mastectomy at the University of Veterinary Medicine, Vienna. Formalin-fixed, paraffin embedded sections (2 µm) of mammary carcinoma tissue were dewaxed and rehydrated. Heat induced epitope retrieval was performed in the Lab Vision PT Module (Thermo Fisher Scientific, Waltham, MA, USA) by incubating slides in ethylenediaminetetraacetic acid (EDTA) buffer at pH 8.0. Further steps were performed in the Lab Vision Autostainer (Thermo Fisher Scientific). Endogenous peroxidase activity was inhibited using Hydrogen Peroxidase Block und Ultra V Block (Thermo Fisher Scientific). For immunostaining Col-1, a monoclonal mouse anti-human CEACAM5 IgG2a antibody (Invitrogen, San Diego, CA, USA) was diluted 1:50 in Ultra Plus Ab Diluent (Thermo Fisher Scientific) and incubated at RT for 30 min. Afterwards slides were incubated with Primary Antibody Enhancer (Thermo Fisher Scientific) for 15 min followed by HRP (horseradish peroxidase) Polymer (Thermo Fisher Scientific) for 20 min at RT. Specific immunoperoxidase staining activity was detected using DAB substrate (Thermo Fisher Scientific). Slides were counterstained with hematoxylin and mounted in Neo Mount (Merck, Darmstadt, Germany). To determine CEAR expression, tissue tumor samples of 10 of the above mentioned mammary cancer patients were selected based on positive Col-1 staining and treated as mentioned above. Non-specific binding was blocked with 5% FCS. Slides were incubated with mouse anti-hnRNP antibody (recognizing isoforms M1 - M4) from Invitrogen, 1:100 in 5% FCS, for 60 min at RT and afterwards with HRP Polymer (Dako, Carpinteria, CA, USA) for 10 min at RT. Further steps were performed as described above and stainings evaluated by microscopy (Tissuegnostics, Vienna, Austria) with a 20X/0.5 or a 40X/1.3 oil objective lens. 

***Cell lines***

Canine mammary carcinoma cell lines CF33 and CF41 and human colon adenocarcinoma cell line HT29 are available from the American Type Culture Collection (ATCC, Rockville, MD, USA; Cat. No.: CRL-6227 for CF33 and CRL-6232 for CF41, Cat. No.: HTB-38 for HT29). All cell lines were cultivated according to the distributor’s protocol in DMEM medium supplemented with 10% heat inactivated fetal calve serum (FCS), 2 mM l-glutamine, penicillin (5,000 U/mL) and streptomycin (100 µg/mL). The canine mammary carcinoma cell lines Sh1b and P114 were a kind gift of Dr. Gerard Rutteman (Department of Clinic Science and Companion Animals, University of Utrecht, The Netherlands) and were maintained in DMEM/F12 supplemented with 10% FCS, 2 mM l-glutamine and 10 µg/mL gentamicin sulfate. Canine oral melanoma cell line TLM-1 was kindly provided by Dr. Jaime F. Modiano (University of Minnesota, Veterinary Clinical Sciences Department, St. Paul, MN, USA). TLM-1 cells were cultured in DMEM containing 10% FCS. Cell lines were kept at 37 °C in a humidified atmosphere of 5% CO2. 

***Western Blotting***

Expression of the canine Col-1 target in canine mammary carcinoma tissue samples and cell lysates was assessed by sodium dodecylsulfate (SDS) gel electrophoresis using reducing conditions followed by Western blot analysis. Briefly, frozen tissue samples were homogenized in 1 ml lysis buffer (10 mM Tris-HCL pH 7.4, 1% SDS). Cells were cultured as described above, washed with ice cold PBS and lysed in 500 µl ice cold lysis buffer (10 mM Tris-HCL pH 7.4, 100 mM NaCl, 1 mM EDTA, 1 mM ethylene glycol tetraacetic acid (EGTA), 1% Triton X-100, 10% glycerol, 0.1% SDS, 0.5% deoxycholate). Lysis buffers contained protease inhibitor cocktail (P8340; Sigma-Aldrich, St. Louis, MO, USA). The lysates were boiled for 5 min and centrifuged at 1000 g. Equal amounts of protein were separated by 10% SDS-PAGE and blotted to polyvinylidene fluoride (PVDF) membranes. To assess equal loading of lysates, we performed reversible Ponceau staining of the membrane using Ponceau S solution (Sigma-Aldrich). 5% dried milk powder in TBS containing 0.1% Tween was used for blocking nonspecific binding sites. Membranes were incubated with Col-1 antibody diluted 1:250 overnight at 4 °C. Anti-mouse IgG2a antibody (SouthernBiotech, Birmingham, AL, USA) was used as isotype control. HRP – conjugated secondary anti-mouse antibody (Abcam, Cambridge, UK) was applied for 1 h at room temperature. For detection of CEA receptor expression in canine and human cell lines, Western blot analysis was performed as described above. Membranes were incubated overnight using either rabbit anti-human CEA receptor (hnRNP M4 long isoform 1) antibody (USB, Swampscott, MA, USA) or mouse anti-hnRNP (M1-M4), each diluted 1:1000. Next, appropriate HRP – conjugated secondary anti-rabbit (Cell Signaling, Danvers, MA, USA) or anti-mouse antibody was applied 1:2000 over a period of 1 h and bound antibodies were detected with the SuperSignal CL-HRP Substrate system (Pierce Biotechnology, Rockford, IL, USA).

***Transient transfection of TLM-1 cells with plasmids encoding CEACAM variants***

Canine TLM-1 cells were cultured in DMEM medium containing 10% calf serum at 37°C in 5% CO2. For transfection, cells were seeded overnight in 6-well plates to reach 50% confluency the next day. Cells were transfected with 4 µg of plasmid encoding canine CEACAM1, canCEACAM23, canCEACAM24, canCEACAM25, canCEACAM28 and canCEACAM30 using the CaPO4 method according the manufacturer's protocol (Stratagene, Agilent Technologies, Santa Clara, CA, USA). To estimate efficiency of transfection, cells were routinely transfected with an empty vector pEGFP-c2 (Clontech Laboratories, Mountain View, CA, USA) encoding green fluorescent protein and analyzed with an inverted microscope (Axioobserver Z1, Zeiss, Oberkochen, Germany) equipped with a high resolution microscopy camera (AxioCam MRc, Zeiss) and a filter set for fluorescein isothiocyanate /enhanced green fluorescent protein (FITC/EGFP) fluorescence. Wells with transfection efficiencies of ≥ 50% were collected for Western Blot analysis. 

***Flow Cytometry***

Cells were trypsinized, counted and resuspended in fluorescence-activated cell sorting (FACS) buffer (1 x PBS supplemented with 2 % heat-inactivated goat serum). For intracellular staining 2 x 105 cells were fixed with formaldehyde for 20 min at RT in the dark, washed once with FACS buffer and afterwards permeabilized with prechilled methanol for 30 min on ice. Next, treated cells were washed once with FACS buffer and incubated with 5 μg Col-1 antibody or isotype control antibody in FACS buffer for 1 h at 4 °C. For surface expression analysis, fixing and permeabilization steps were omitted. Stained cells were again washed with FACS buffer, followed by incubation of FITC – conjugated anti-mouse IgG (Jackson ImmunoResearch Lab, West Grove, PA, USA) for 30 min at 4 °C. After washing the pellet was resuspended in 300 μl FACS buffer and 10,000 events were counted using a FACSCalibur flow cytometer (Becton Dickinson, San Jose, CA, USA). Data analysis was performed via CellQuest software. 

*****Cell Viability Assay***

Tumor cell proliferation was analyzed by a modified tetrazolium dye assay (EZ4U Cell Proliferation Assay; Biomedica Group, Vienna, Austria) based on the ability of living cells to reduce tetrazolium salts into formazan derivates. Cells were seeded in 96-well plates at 2 x 104 cells per well and allowed to adhere overnight under standard culture conditions. 1 µg Col-1 or isotype control antibody per 200 µl medium was applied over a period of 24 h and 48 h. Control wells were given media alone or treated with 0.9% Triton X-100 for 10 min prior to addition of the modified tetrazolium dye. The quantity of reduced formazan is directly proportional to living cells. Therefore absorbance was measured after 1 h incubation at 450 nm with 620 nm as a reference with a 96-well plate reader. All experiments were done in triplicates. 

***Signaling Pathway Assay***

Phosphorylation assays were performed in CF33 and HT29 cells after 24 h serum starvation in DMEM medium containing 0.1% FCS. After culturing for 24 h, cells were then treated with either fresh medium containing 0.1 % serum alone (control) or with 2 µg/mL Col-1 antibody in the same medium for the time periods indicated. Next, cell monolayers were washed with ice-cold PBS, lysed and subjected to Western blot analysis as described above. The phosphorylation patterns were determined by probing the blots with anti-AKT, anti-phospho AKT (targeting serin 473), anti-phospho AKT (targeting threonine 308), anti-phospho p44/42 MAPK (all from Cell Signaling) and anti-MAPK antibody (Santa Cruz Biotechnology, Santa Cruz, CA, USA) all diluted 1:1000, followed by HRP – conjugated anti-rabbit antibody before detection using the ECL method (Amersham, Arlington Heights, IL, USA). Densitometric analyses were carried out using ImageJ software. 

***Sequence and phylogenetic analyses***

Phylogenetic analyses based on nucleotide and amino acid sequence alignments were conducted in “Molecular evolutionary genetics analysis” (MEGA5) [45] [46]. The Maximum Likelihood method based on the JTT matrix-based model (protein sequences) [47] or based on the Tamura-Nei model (DNA sequences) [48], with bootstrap testing (500 replicates) was applied. The multiple amino acid sequence alignment shown in Figure 1 and in Supplementary Figure 1 were performed with ClustalW at NPS@ (Network Protein Sequence @nalysis;http://npsa-pbil.ibcp.fr/) and verified using MEGA5.

***Similarity of CEACAMs and CEAR between Humans and Dogs***

Human and canine CEACAMs and CEAR proteins from ncbi (http://www.ncbi.nlm.nih.gov/protein) were aligned using BLAST (Basic Local Alignment Tool; http://blast.ncbi.nlm.nih.gov/Blast.cgi). Sequences stored under the accession number NP_001091026 (canine CEACAM1), NP_001091021 (canine CEACAM23), NP_001091023 (canine CEACAM24), ABL76056 (canine CEACAM25), ABL76057 (canine CEACAM28), NP_001091022 (canine CEACAM30), NP_005959.2 (human CEAR), XP_854270.1 (canine CEAR), NP_001178152.1 (bovine CEAR), NP_001103381.1 (Rat CEAR), NP_084080.1 (Mouse CEAR), NP_001026103.1 (Chicken CEAR), AAI34826.1 (Xenopus CEAR), NP_001133640.1 (Salmon CEAR) and XP_683303.4 (Zebrafish CEAR) were used for homology alignment searches. 

## Results

***Phylogenetic comparison of human and canine CEACAMs***

Our BLAST analysis across the whole sequences revealed amino acid sequence identities ranging from 42 - 59% and similarities between 55% and 72% between human CEACAM5 and canine CEACAM1-related molecules (Fig. 1a). Phylogenetic analysis comparing N domain exon nucleotide sequences of human and canine CEACAM1-related CEACAMs and CEACAM19, respectively, indicated that CEACAM1-related CEACAMs between the different species are more closely related than CEACAM1-related and other CEACAM types within the same species. The results were corroborated by three independent methods, Maximum likelihood (ML), Maximum parsimony (MP) using MEGA5 and Neighbor-joining (NJ) using ClustalW (data not shown) (Fig. 1b). Amino acid sequences of mature CEACAM1-related N domains without leader peptide were aligned using ClustalW. Especially the N domain of human CEACAM5 contains multiple regions that are highly similar to regions of canine CEACAM1-related CEACAM N-domains (Fig. 1c).

**Figure:**
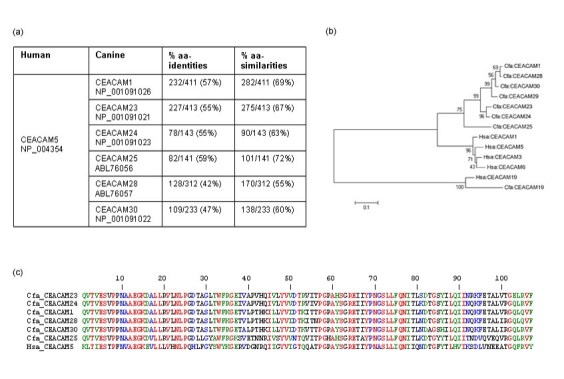
**Fig. 1 **Comparison of human CEA (CEACAM5) with canine CEACAMs. **(a)** BLAST analysis across the whole sequences of human CEACAM5 and canine CEACAM1-related molecules revealed amino acid sequence identities ranging from 42 - 59% and similarities between 55% and 72%. **(b) **A phylogenetic tree was constructed using MEGA5 from N domain exon nucleotide sequences of selected human and canine CEACAM1-related CEACAMs and CEACAM19, respectively. It shows that CEACAM1-related CEACAMs between the different species are more closely related than CEACAM1-related and other CEACAM types within the same species. **(c) **Especially the N domain of human CEACAM5 contains multiple regions that are highly similar to regions of canine CEACAM1-related CEACAM N domains. Amino acid sequences of mature CEACAM1-related N domains without leader peptide were aligned using ClustalW. The following color code was used: red, identical amino acids; green, strongly similar amino acids; blue, weakly similar amino acids; black, non-conserved amino acids.

***Immunoreactivity of Col-1 in canine mammary cancer cells***

In 23 out of 30 investigated canine mammary cancer patients Col-1 reactive tumor cells could be detected via immunohistochemical analysis, revealing individually varying staining intensities. Figure 2a shows typical examples of positive immunoreactivity which was loca lized predominantly in the cytoplasm of tumor cells. 

The presence of a Col-1 binding signal in canine mammary cancer cells was confirmed by Western blot analysis using reducing conditions. In cell lysates of CF33 and CF41 cells, but not in Sh1b and P114 cells, we observed a specific protein band at 60 kDa, whereas staining with isotype control antibody remained negative (Fig. 2b). The result was confirmed by blotting lysa tes from spontaneous mammary carcinoma of canine patients that had proven positive for Col-1 reactivity by immunohistochemistry (Fig. 2c). In 3 out of 4 samples from dog patients, Col-1 binding was revealed at 60 kDa and weaker at 120 kDa. In dog 4, like in human HT29 colon cancer cells (Fig. 2b), Col-1 binding was observed at approximately 180 kDa corresponding to the molecular weight of human CEACAM5. Next we investigated whether Col-1 would recognize important members of the canine CEACAM family. We selected canine TLM-1 melanoma cells as host for transfection and optimal species-specific expression of canine CEACAM1, -23, -24, -25, -28 and -30, all belonging to the canine CEACAM1 family. Col-1 binding was only found in cells transfected with human CEACAM5 but not in cells transfected with any of the canine CEACAMs (Fig. 2d). These data indicate that Col-1 does not crossreact with the above-mentioned canine CEACAM isoforms. 

**Figure:**
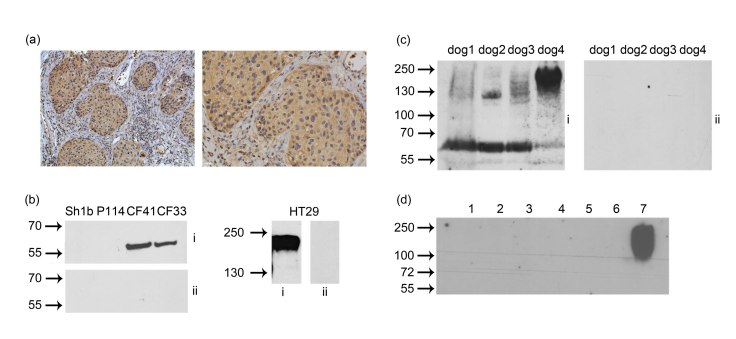
**Fig. 2** Col-1 is immunoreactive in canine mammary cancer. **(a)** Sections of canine tumor specimens were stained immunohistochemically with Col-1 antibody. The same region of the tumor tissue sample was evaluated by microscopy with a 20X/0.5 and a 40X/1.3 oil objective lens. **(b)** Western blot analysis revealed a Col-1 immunoreactive signal in canine mammary carcinoma cells CF33 and CF41 at approximately 60 kDa. P114 and Sh1b remained negative. Human colon cancer cells HT29 served as positive control showing a strong Col-1 signal at 180 kDa. **(c) **Tumor tissue samples from four different canine mammary carcinoma patients also displayed a Col-1 reactive protein band. Samples from dog 1 to 3 showed positive signals at 60 kDa and 120 kDa, respectively, whereas the protein detected in the sample from dog 4 had an apparent molecular weight of approximately 180 kDa. **(d)** Total lysates derived from TLM-1 cells transfected with canine CEACAM23, CEACAM24, CEACAM25, CEACAM28, CEACAM30, CEACAM1 (lane 1-6) or human CEACAM5 cDNA (lane 7) were blotted. Col-1 did not bind to any of the tested canine variants, but only to cells transfected with human CEACAM5 cDNA. Murine monoclonal antibody Col-1 (i) or IgG2a isotype control antibody (ii) were detected by HRP – conjugated anti-mouse antibody.

***Intracellular binding in flow cytometry and no effects on growth by Col-1***

To verify the expression of the epitope recognized by Col-1 in canine cancer cell lines we performed flow cytometry assays. In contrast to human HT29 cells which showed strong membranous Col-1 binding, all tested canine cells remained negative (Fig. 3a). However, specific cytoplasmic Col-1 staining was revealed after permeabilizing CF33 and CF41, and weak in Sh1b and P114 cells (Fig. 3b). Staining with the isotype control antibody remained negative in all intracellular and surface expression assays. In agreement with the Western blotting experiments these results confirmed that Col-1 binds to a specific target in canine mammary carcinoma cell lines.

**Figure:**
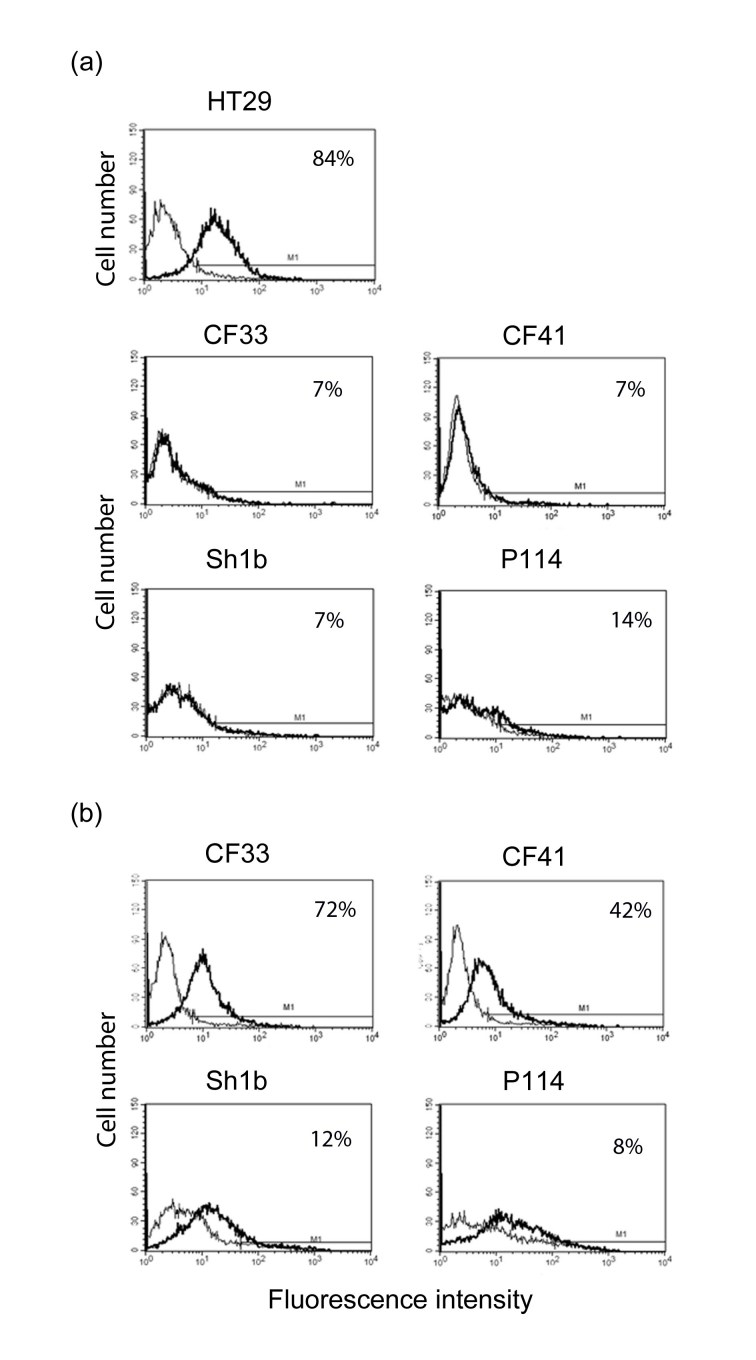
**Fig. 3** Binding of Col-1 to canine or human mammary cancer cell lines determined by flow cytometry. **(a) **HT29 showed high surface expression levels as expected. None of the canine cancer cells (CF33, CF41, Sh1b and P114) showed any extracellular Col-1 binding. **(b) **Permeabilization of canine cells revealed a strong intracellular signal in CF33 and CF41, and weak Col-1 binding in Sh1b and P114. Cell line were stained using mAb Col-1 (bold line) or isotype matched control antibody (fine line) followed by FITC-conjugated secondary antibody.

The potency of Col-1 to affect proliferation of canine and human tumor cells in vitro was evaluated. CF33 and HT29 were selected due to the highest staining intensity in Western blot and flow cytometry analysis. When Col-1 was applied over a period of 24 h and 48 h, neither in canine nor in human cells significant effects on cell proliferation could be achieved as compared to the isotype control antibody (data not shown).

***Differences in AKT phosphorylation between human and canine cells***

Our results of the phosphorylation assay revealed that phospho-AKT levels in human HT29 cells were undetectable after serum starvation, but upregulated after both 7.5 and 15 min of Col-1 treatment. In contrast, phospho-AKT levels in canine CF33 cells remained elevated even after prolonged serum starvation and were subsequently downregulated in the presence of Col-1. Two different antibodies against phospho-AKT were used, both showed similar results, indicating phosphorylation at multiple locations. Total AKT levels were unaffected in both cell lines. Under the same conditions, total MAPK as well as MAPK phosphorylation levels remained unchanged in HT29 as well as in CF33 cells (Fig. 4). 

**Figure:**
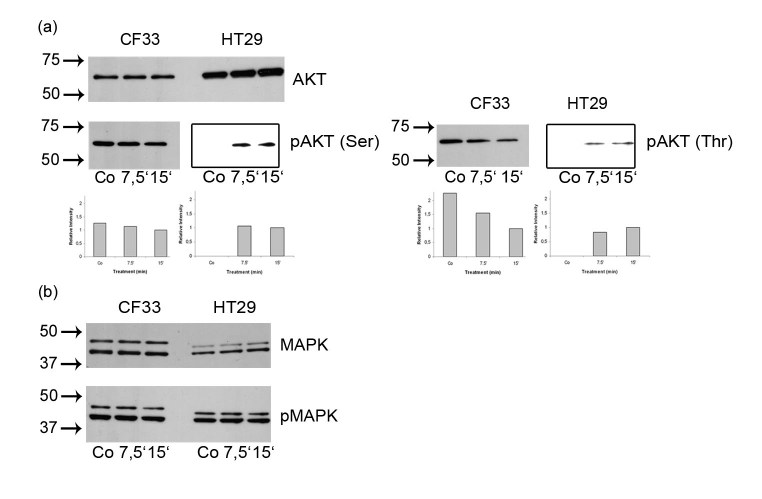
**Fig. 4 **Effects of Col-1 treatment on phosphorylation pattern of AKT and MAPK pathways in human versus canine cancer cells. **(a)** In human HT29 colon cancer cells Col-1 treatment after 7.5 min and 15 min induced AKT phosphorylation detected by two different anti-phosphoAKT antibodies (pAKT(Ser473), pAKT(Thr308)). In contrast, a slight decrease of AKT phosphorylation was observed in CF33 cells with both antibodies. Results of densitometric analysis using ImageJ software are given below blots. The signal intensities at 48 h incubation were used as a reference. **(b)** Phosphorylation levels of both MAPK (ERK1 and ERK2) isoforms were unaffected in both cell lines by Col-1 treatment.

***High similarity of the human and canine carcinoembryonic antigen receptor***

Soluble human CEACAM5 binds to a specific CEA receptor (CEAR), which is a member of the hnRNP family. By BLAST analysis we found a homologue to human CEAR in the genome of dog, and exemplarily for other vertebrates, in bovine, rat, mouse, chicken, xenopus, salmon and zebrafish. The amino acid identity of CEAR in mammalians was above 98%, and 99% between human and dog, respectively (Fig. 5a). Further, homologies of CEARs among chicken, xenopus, salmon and zebrafish ranged lower, from 50 to 73% amino acid identities. The whole sequence alignments are given in supplementary figure 1. Based on these data a phylogenetic tree was constructed for CEAR with MEGA5 (Fig. 5b) and approved by ClustalW (data not shown), which indicates that the interspecies relationship of this molecule closely follows the principle of evolution: Bovine and canine CEAR are more closely related to human CEAR than any other depicted species (Fig. 5b). Immunoblots with anti-human CEAR antibodies directed against two different isoforms identified not only human CEAR in HT29 cells but also canine CEAR in all canine mammary carcinoma cell lines tested (Fig. 5c). Next, tissue sections of 10 different Col-1 positive canine mammary cancer patients were immunostained for CEAR. All 10 tissue samples showed positive immunoreactivity with anti-human hnRNP (M1-M4) antibody, mainly in the nuclear region of the tumor cells. In Figure 5d, CEAR staining is shown in samples from the same two patients depicted in Figure 2a. 

**Figure:**
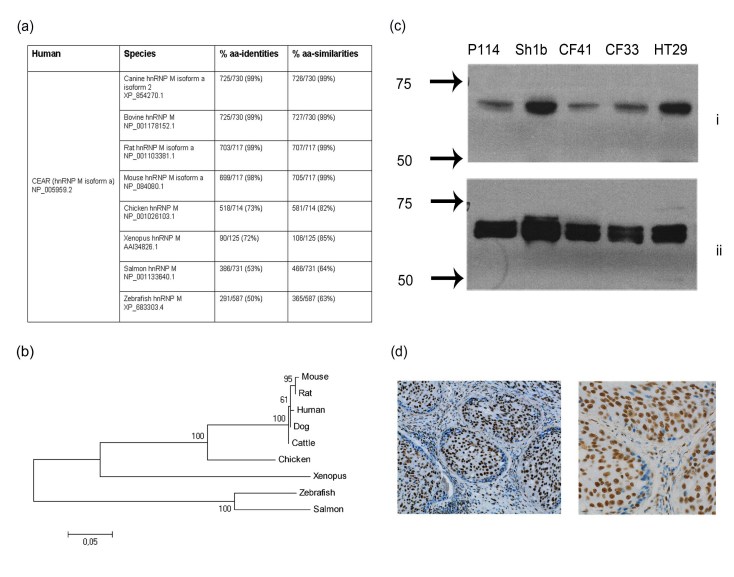
**Fig. 5** CEA-receptor (CEAR) is phylogenetically highly conserved and expressed in canine mammary carcinoma. **(a)** BLAST analysis revealed more than 98% homology in selected mammalians, and 99% amino acid sequence homology between human and canine CEAR. **(b)** Using MEGA5 a phylogenetic tree was constructed. It shows that within mammalians canine CEAR is most closely related to human CEAR; other species like bird, frog and fish range lower**.** **(c)** Two different antibodies specific for human CEAR, rabbit anti-CEAR long antibody (i) as well as mouse anti-hnRNP antibody (ii) identified a CEAR-specific signal in canine P114, Sh1b, CF41 and CF33 cell lysates at ~70 kDa similar as well as in human HT29 cancer cell lysates serving as a positive control. **(d)** Immunostaining of primary lesions of canine mammary cancer patients which were positive for Col-1 staining was mainly observed in the nuclear region of tumor cells with anti-hnRNP antibody. The same region of the tumor tissue sample was evaluated by microscopy with a 20X/0.5 and a 40X/1.3 oil objective lens.

## Discussion 

Using Col-1 monoclonal anti-CEACAM5 antibody we have previously developed a mimotope-based anti-CEACAM5 vaccine for the treatment of human carcinoma. Mimotope vaccination prevented the growth of transplanted Meth-A/CEA tumor cells in a BALB/c mouse model [31]. Inspired by the concept of comparative oncology we intended to test the applicability of the mimotope vaccine in canine cancer patients. Therefore, we focused here on the canine target molecule of Col-1 being a putative CEACAM homologue. We soon realized the complexity of this approach because the CEACAM protein family exhibits an extreme diversity within different species in vertebrates [44]. This is connected to the fact that they play a role in multiple processes, including homotypic and heterotypic adhesion, cellular differentiation and as pathogen receptors. In most mammalian orders orthologs to CEACAM1 exist [49]. The primordial CEACAM1 gene was subject to species-specific gene amplification leading to groups of CEACAM1-related genes which vary considerably between mammalian species. In humans CEACAM5 is a glycosylphosphatidylinositol (GPI)-anchored molecule of the CEACAM1-related CEACAMs which can be cleaved by GPI-phospholipase D (GPI-PLD), canine CEACAM1-related CEACAMs are mostly ITAM- (Immunoreceptor Tyrosine-based Activation Motif-) bearing transmembrane proteins encoded by at least five functional genes [50] [51]. Interestingly, in the canine genome a CEACAM5 equivalent could not be found [44].

In accordance with previous immunohistochemistry studies [35] [36] [37] and being aware of the lack of a known canine CEACAM5 ortholog, our immunohistochemical analysis showed specific Col-1 staining in 23 of 30 canine mammary cancer patients. When we probed lysates of 4 primary mammary tumors we found that only one of the samples showed a specific Col-1 signal at the expected molecular weight of 180 kDa of the human CEACAM5 molecule. The human 180 kDa CEACAM5 is built up by 6 immunoglobulin domains and additionally decorated by glycans of the N-glycosylated complex which contribute to about half of the molecule´s mass [30] [52]. As in 3 of the 4 tested canine mammary cancer samples faster migrating bands were detected at ~60 and ~120 kDa by Col-1, suggestive that they could represent fragments of the canine 180 kDa CEACAM molecule. Also in the canine mammary carcinoma cell lines CF33 and CF41 60 kDa molecules were specifically identified by Col-1.

Our phylogenetic search indicated that in the canine genome CEACAM1-related CEACAMs would be most closely related to human CEACAM5. However, a transfection experiment indicated that most widely expressed canine CEACAM1-related molecules, namely CEACAM1, -23, -24, -25, -28 or -30, were not recognized by Col-1. Having used canine TLM-1 melanoma cells for transfection we exclude that the negative binding was due to incorrect species-specific post-translational modification.

Specific expression of the Col-1 target was further demonstrated by flow cytometry where, in contrast to human HT29 cells with membranous and cytoplasmic expression, the antigen was detected only intracellularly in the canine mammary CF41 and CF33 cells. This is in accordance with the present immunohistochemistry in canine mammary tumor samples, and previous reports on cytoplasmic CEA staining in human head and neck [40] and colorectal cancer [53]. As it has been repeatedly demonstrated that also intracellular targets can be approached by natural and engineered antibodies [54] [55] [56], this fact would not contradict the principle of antibody-mediated targeting.

To investigate the potential functional relevance of Col-1 targeting in human or canine cancer we performed growth inhibition and signal transduction assays. Col-1 treatment did neither induce growth inhibition in canine CF33 mammary cancer cells nor in human HT29 colon cancer cells. In line with that we previously ascribed the cytotoxic effect of a Col-1 mimotope vaccine to antibody-mediated tumoricidity [31]. Our data from signal transduction assays indicate that Col-1 targeting of human and canine cells leads to divergent effects on AKT phosphorylation, which was increased in human HT29 cells but slightly decreased in canine CF33 cells. Therefore, thorough antibody validation prior to use across species is mandatory. The AKT signaling pathway plays a key role in multiple cellular processes such as glucose metabolism, cell proliferation, apoptosis, transcription and cell migration. Possibly, the biological effect of Col-1 targeting may also depend on the activation state of the AKT signaling pathway in the different species. MAPK pathway, likewise being involved in multiple biological effects, such as regulation of gene expression, mitosis, proliferation and cell survival, remained unaffected by Col-1 treatment in both human and canine cancer cells.

A homolog of heterogeneous nuclear protein M4 (hnRNP M4) termed CEA-receptor (CEAR) has previously been shown on the membrane as well as in the cytoplasm of human colon cancer cells [57]. The fact that CEAR may co-localize with CEA is suggestive for signaling function of this complex [16]. We report here for the first time that CEAR is much higher conserved among mammalians than in other vertebrates, like bird, frog, and fish. With 99% amino acid identity, conservation of CEAR is especially pronounced in the human and canine species. Further, CEAR is highly expressed in human as well as canine mammary carcinoma. Using anti-human CEAR antibody, it could not only be detected in all tested canine cancer cell lines but also in 10 out of 10 tissue samples from spontaneous canine mammary cancers. As mentioned above no CEACAM5 equivalent with the potential to act as soluble form has been found in the dog genome so far. Our data show that anti-human CEACAM5 antibody Col-1 specifically detects 180, 120 and 60 kDa molecules in canine cancer cells which are most likely non-related to the canine CEACAM1-related proteins. Future studies will reveal whether these molecules may adapt ligand function to the canine CEAR.

The fact that the inter-species heterogeneity of CEACAM molecules in general was greater than expected was in striking contrast to the high conservation of the CEA receptor molecule between humans and dogs. Our initial expectations that spontaneous mammary carcinoma in dogs could serve as a model for human breast cancer with respect to CEACAM targeting could thus, discordant to the intriguing comparative oncology concept, not be fulfilled. Though, the finding that CEAR is highly homologous between humans and dogs, and specifically expressed in canine mammary cancer might open up new avenues for anticancer developments based on the comparative oncology strategy.

## Acknowledgments 

The authors want to thank Philipp Starkl for helpful discussions. Erika Jensen-Jarolim is designated as corresponding author of this manuscript.

## Funding information

This study was supported by the RotePfote (http://www.rotepfote.at) grant #FA648A1301. MW is a recipient of a Von Fircks-Scholarship of the University of Veterinary Medicine, Vienna. JS is supported by the CCHD program of the Austrian Science Fund FWF (#APW01205FW).

## Competing interests 

The authors have declared that no competing interests exist. 

**Figure:**
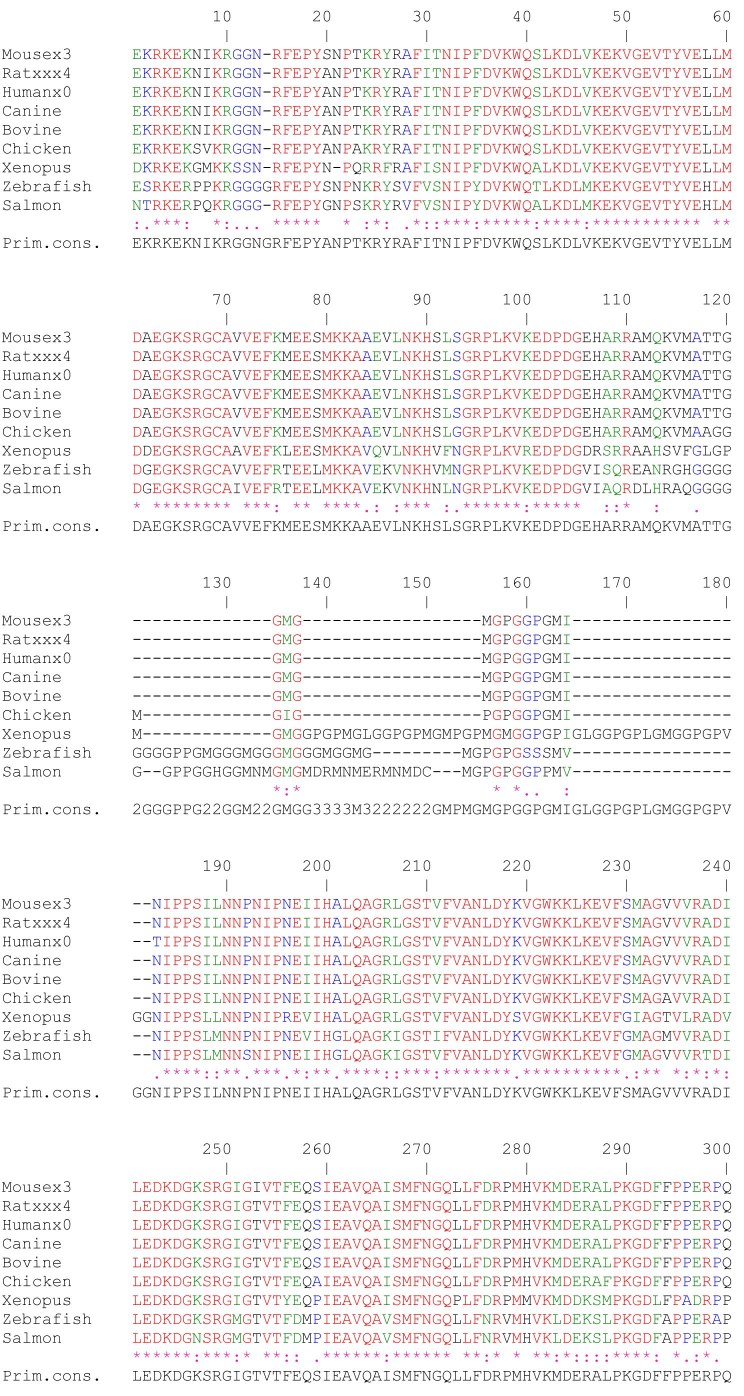

**Figure:**
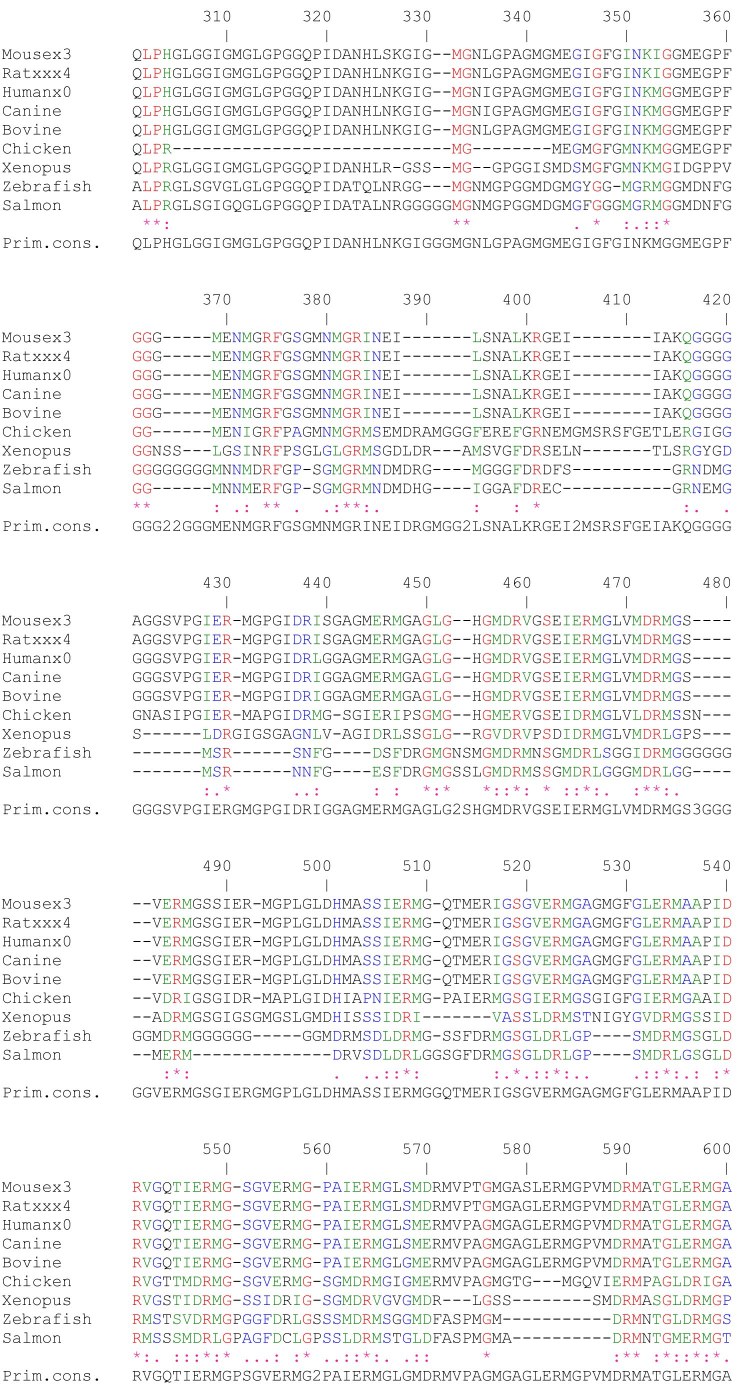

**Figure:**
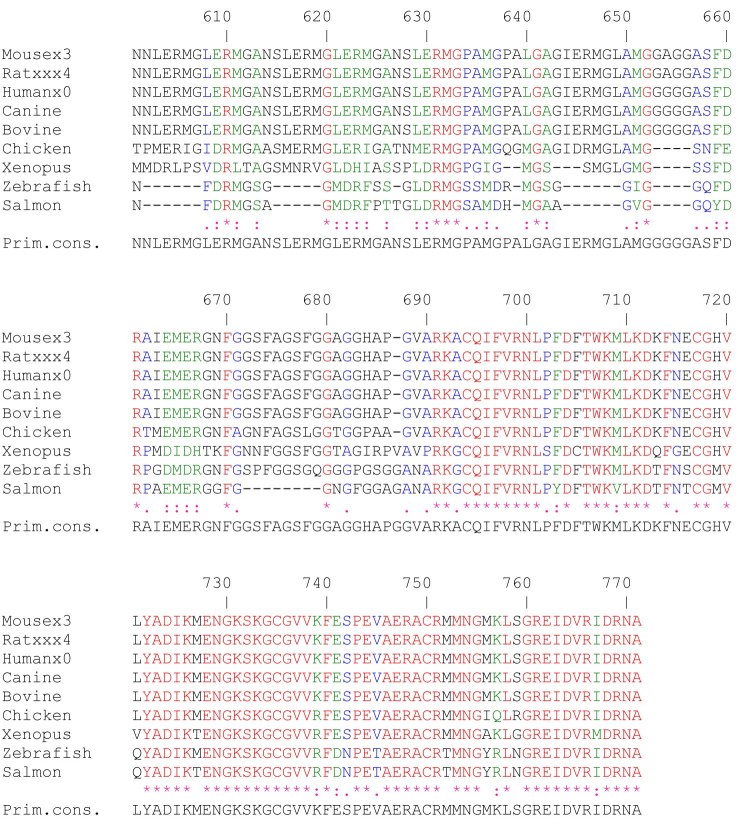
**Supplementary Fig 1**Multiple amino acid sequence alignments on CEARs of different species using ClustalW. The following color code was used: red, identical amino acids; green, strongly similar amino acids; blue, weakly similar amino acids; black, non-conserved amino acids.
